# Crystal structure of human peptidylarginine deiminase type VI (PAD6) provides insights into its inactivity

**DOI:** 10.1107/S2052252524002549

**Published:** 2024-04-24

**Authors:** Fanomezana M. Ranaivoson, Rieke Bande, Isabell Cardaun, Antonio De Riso, Annette Gärtner, Pui Loke, Christina Reinisch, Prasuna Vogirala, Edward Beaumont

**Affiliations:** a Protein Sciences Department, Evotec (United Kingdom), 95 Park Drive, Abingdon OX14 4RY, United Kingdom; b Assay Development Department, Manfred Eigen Campus, Evotec (Germany), Essener Bogen 7, 22419 Hamburg, Germany; c In vitro Biology Department, Manfred Eigen Campus, Evotec SE, Essener Bogen 7, 22419 Hamburg, Germany; d Chemistry Department, Evotec (United Kingdom), 95 Park Drive, Abingdon OX14 4RY, United Kingdom; University of Michigan, USA

**Keywords:** mammalian fertilization, cytoplasmic lattices, human peptidylarginine deiminase VI, protein structures, PAD6

## Abstract

Human peptidylarginine deiminase isoform VI (PAD6), which is predominantly limited to cytoplasmic lattices in the mammalian oocytes in ovarian tissue, is essential for female fertility. It belongs to the peptidylarginine deiminase (PAD) enzyme family that catalyzes the conversion of arginine residues to citrulline in proteins. In contrast to other members of the family, recombinant PAD6 was previously found to be catalytically inactive. We sought to provide structural insight into the human homologue to shed light on this observation. We report here the first crystal structure of PAD6, determined at 1.7 Å resolution. PAD6 follows the same domain organization as other structurally known PAD isoenzymes. Further structural analysis and size-exclusion chromatography show that PAD6 behaves as a homodimer similar to PAD4. Differential scanning fluorimetry suggests that PAD6 does not coordinate Ca^2+^ which agrees with acidic residues found to coordinate Ca^2+^ in other PAD homologs not being conserved in PAD6. The crystal structure of PAD6 shows similarities with the inactive state of *apo* PAD2, in which the active site conformation is unsuitable for catalytic citrullination. The putative active site of PAD6 adopts a non-productive conformation that would not allow protein–substrate binding due to steric hindrance with rigid secondary structure elements. This observation is further supported by the lack of activity on the histone H3 and cytokeratin 5 substrates. These findings suggest a different mechanism for enzymatic activation compared with other PADs; alternatively, PAD6 may exert a non-enzymatic function in the cytoplasmic lattice of oocytes and early embryos.

## Abstract 2

The human peptidylarginine deiminase type VI (PAD6) is essential in oocyte and embryonic development as a component of the supramolecular assemblies called cytoplasmic lattices. The crystal structure presented here suggests PAD6 assembles as a dimer resembling other PADs, albeit with compromised abilities to bind Ca^2+^ and substrates. This aligns with existing *in vitro* data which indicate an enzymatically inactive isoform of PAD.

## 1. Introduction

PAD enzymes play crucial roles in various cellular processes through their ability to convert arginine residues on proteins to citrulline in a Ca^2+^-dependent manner (Arita *et al.*, 2004 ▸; Slade *et al.*, 2015 ▸). Post-translational citrullination is involved in important physiological processes, such as skin keratinization, neuron insulation and inflammation, or in development (Senshu *et al.*, 1999 ▸; Boggs *et al.*, 1999 ▸; Suzuki *et al.*, 2003 ▸; Wright *et al.*, 2003 ▸). In mammals, five PAD isoforms have been identified (PAD1–PAD4 and PAD6). They have been shown to be expressed in a tissue-specific manner with distinct substrate specificity (Vossenaar *et al.*, 2003 ▸). PAD6 is the most recently characterized member of the PAD family and is specifically expressed in oocytes (Wright *et al.*, 2003 ▸). It was found to play a significant role in oocyte maturation and the development of the fertilized oocyte beyond the two-cell stage (Esposito *et al.*, 2007 ▸; Yurttas *et al.*, 2008 ▸). Female *Pad6* knock-out mice are infertile due to defects in early embryo development (Esposito *et al.*, 2007 ▸). Similarly, women with PAD6 mutations have been reported to experience premature embryonic arrest and infertility (Xu *et al.*, 2016 ▸; Liu *et al.*, 2021 ▸; Begemann *et al.*, 2018 ▸). On the cellular level, PAD6 is found within keratin-containing cytoplasmic lattices (CPLs) in mice (Wright *et al.*, 2003 ▸). CPLs are proposed to serve as a storage depot for maternal proteins important for embryogenesis (Yurttas *et al.*, 2008 ▸; Jentoft *et al.*, 2023 ▸). Keratins, particularly cytokeratin 5 and 6, are known to be major constituents of cytoskeletal lattices (Schwarz *et al.*, 1995 ▸). Moreover, keratins are suspected to be targets of citrullinating enzymes in keratinocytes (Nachat *et al.*, 2005 ▸). Although indirect evidence supports citrullinating activity of PAD6 in oocytes, as indicated by immunostaining of citrullinated protein in wild-type versus PAD6 knock-out mice (Esposito *et al.*, 2007 ▸), no direct *in vitro* evidence for such activity has been observed. *In vitro* methods used for measuring the activity of other PAD isoforms using l-arginine-based synthetic and peptide substrates have not provided conclusive evidence for PAD6 activity (Raijmakers *et al.*, 2007 ▸; Taki *et al.*, 2011 ▸). The human PAD4 isoform has been extensively investigated in terms of its structure–function relationships through examination of crystal structures in *apo*, *holo* and ligand-complexed forms, as well as the analysis of enzyme mutants (Arita *et al.*, 2004 ▸, 2006 ▸; Liu *et al.*, 2011 ▸; Lewis *et al.*, 2015 ▸; Lee *et al.*, 2017 ▸). PAD4 has a dimeric structure where the N- and C-terminal regions are responsible for Ca^2+^ binding and catalytic activity, respectively. The binding of Ca^2+^ to *apo*-PAD4 induces a conformational transition into its catalytically active state (Arita *et al.*, 2004 ▸). The present study provides insights into the structure and function of PAD6, compared with PAD4 and other PAD enzymes, and will facilitate future studies on the role of PAD6 in female fertility.

## 2. Materials and methods

### 2.1. Protein expression and purification

The synthetic gene of the recombinant human PAD6 (V2-P694 S10E S446E, Fig. S1 of the supporting information) with a TEV protease-cleavable 6×His-GST tag fused to its N-terminus was codon-optimized for mammalian expression and cloned into the pcDNA3.4 vector using TOPO cloning strategy (GenScript). The resulting construct was used for transient transfection in HEK cells using PEI-MAX (Sigma–Aldrich) and FreeStyle media (Gibco). To purify PAD6, the cell pellet was resuspended in lysis buffer containing 20 m*M* Tris–HCl pH 7.5, 200 m*M* NaCl, 10% glycerol, 1 m*M* TCEP, 2 m*M* MgCl_2_, 10 U ml^−1^ benzonase and complete protease inhibitor tablets (Roche Applied Science). Cells were lysed by sonication, 5 cycles of 20 s on at 40% amplitude and 20 s off on ice, and the clear lysate was obtained by centrifugation. The His-GST tagged protein was bound to gluta­thione Sepharose 4FF resin (Cytiva) for 2 h at 4°C with gentle rocking rotation. PAD6 was washed with 20 m*M* Tris–HCl pH 7.5, 200 m*M* NaCl, 10% glycerol, 1 m*M* TCEP and eluted with 20 m*M* Tris–HCl pH 7.5, 200 m*M* NaCl, 10% glycerol, 1 m*M* TCEP, 10 m*M* reduced gluta­thione. The tagged protein was treated with TEV protease (with a ratio of 1 mg TEV per 20 mg PAD6) overnight at 4°C, and the untagged protein was purified with HisTrap FF (Cytiva) reverse nickel-affinity chromatography followed by size-exclusion chromatography on Superdex 200 with 20 m*M* Tris–HCl pH 7.5, 200 m*M* NaCl, 1 m*M* TCEP before concentrating to 3.2 mg ml^−1^.

The recombinant human PAD4 construct (Fig. S1), used for DSF and aSEC, was adapted from Muth *et al.* (2017 ▸). Briefly, the GST-PAD4 was expressed by autoinduction in ZYM-5052 media (Teknova) in BL21(DE3) pLysS over 18 h at 18°C. Cells were lysed in 20 m*M* Tris–HCl pH 7.5, 200 m*M* NaCl, 10% glycerol, 1 m*M* TCEP, protease inhibitor, 10 U ml^−1^ benzonase, 2 m*M* MgCl_2_. The GST-tagged protein was bound to a GSTrap 4B column (Cytiva) and eluted with 20 m*M* Tris–HCl pH 7.5, 200 m*M* NaCl, 10% glycerol, 1 m*M* TCEP, 10 m*M* reduced gluta­thione. The protein was further purified using a Resource Q column (Cytiva) before tag removal using HRV 3C protease (PreScission) incubation overnight at 4°C (with a ratio of 1 mg HRV 3C protease per 40 mg PAD4). The untagged protein was further purified by reverse GSTrap affinity chromatography followed by size-exclusion chromatography using a Superdex 200 with 20 m*M* Tris–HCl pH 7.5, 400 m*M* NaCl, 0.5 m*M* TCEP before concentrating to 3.1 mg ml^−1^.

### 2.2. Analytical size-exclusion chromatography

The oligomerization of PAD6 in solution was examined by analytical size-exclusion chromatography on a Superdex 200 increase 5/150GL (Cytiva). The column was equilibrated with 20 m*M* Tris–HCl pH 7.5, 200 m*M* NaCl, 1 m*M* TCEP and calibrated using a set of molecular weight protein standards (Bio-Rad) composed of bovine thyroglobulin (670 kDa), bovine gamma globulin (158 kDa), chicken ovalbumin (44 kDa) and horse myoglobulin (17 kDa). The PAD6 sample (1 mg ml^−1^) and the standards were all run at 0.3 ml min^−1^.

### 2.3. Differential scanning fluorimetry

Differential scanning fluorimetry (DSF) was performed using SYPRO Orange (Sigma) as the shift reporter dye. Briefly, 4 µ*M* of protein was incubated in buffer (10 m*M* Tris–HCl pH 7.5, 200 m*M* NaCl with and without 10 m*M* of CaCl_2_) for 30 min on ice. SYPRO Orange dye (Sigma) was diluted to 2× final concentration from 5000× stock. The reactions were monitored in real time (Stratagene MX3005P; excitation, 490 nm; emission, 575 nm) from 25 to 95°C with a rate of change of 0.5°C min^−1^.

### 2.4. Determination of PAD6 crystal structure

#### 2.4.1. Crystallization

The purified PAD6 (3.2 mg ml^−1^) crystallized by vapour diffusion at 18°C using 15–18% PEG 3350, 200–300 m*M* NaBr, 0.1 *M* bis-tris propane pH 6.5 as precipitant. Crystals appeared over two days after mixing equal volumes of protein sample and precipitant and matured to their final sizes after ∼15 days. Crystals were then harvested, cryo-protected with reservoir solution supplemented with 25%(*v*/*v*) PEG 200 and flash-cooled in liquid nitro­gen for diffraction data collection.

#### 2.4.2. Diffraction data collection and processing, structure determination and refinement

Diffraction data were collected at the I03 beamline at Diamond Light Source (Harwell, United Kingdom). A complete dataset was collected from an individual crystal under a cryogenic stream at 100 K at a wavelength of 0.98 Å and processed using the automated pipeline *autoPROC* (Vonrhein *et al.*, 2011 ▸) that executes *XDS* (Kabsch, 2010 ▸), *POINTLESS* (Evans, 2006 ▸) and *AIMLESS* (Evans & Murshudov, 2013 ▸) of the *CCP*4 suite (Winn *et al.*, 2011 ▸), as well as the *STARANISO* module (Tickle *et al.*, 2018 ▸). The PAD6 structure was solved by molecular replacement using a model generated by *AlphaFold* [accession No. AF-Q6TGC4-F1 (Jumper *et al.*, 2021 ▸)]. This model was then refined using *BUSTER* (Bricogne *et al.*, 2017 ▸) and manually corrected using *Coot* (Emsley *et al.*, 2010 ▸). The data collection and refinement statistics of the final model are shown in Table 1 ▸.

### 2.5. Detection of citrullinated proteins by 4-azido­phenyl glyoxal cyclo­addition on membranes (on-blot assay)

100 µg ml^−1^ histone H3 (Abcam) or 20 µg ml^−1^ cytokeratin 5 (Abcam) was incubated with 200 n*M* PAD6 or 100 n*M* PAD4 (Cayman) ± 50 µ*M* GSK484 (Sigma) in citrullination buffer (50 m*M* Tris–HCl pH 7.6, 1 m*M* CaCl_2_, 200 m*M* NaCl_2_, 2 m*M* DTT) in a 384-well Nunc MaxiSorp plate (Thermo Scientific) for 3 h at 37°C and 300 r.p.m. shaking. The reaction was stopped by the addition of EGTA, pH 8.0, to a final concentration of 50 m*M*. The citrullination was detected using the alkyne-biotin based method as described by Hensen *et al.* (2015 ▸). The blots were scanned using the LiCor Odyssey CLx and quantification of protein bands was performed with the integrated *Image Studio* software.

### 2.6. Detection of citrullinated histone H3 by enzyme-linked immunosorbent assay

Enzyme-linked immunosorbent assay (ELISA) assay described by Verheul *et al.* (2018 ▸) was adapted as follows: 1 µg ml^−1^ histone H3 (Abcam) was incubated with 200 n*M* PAD6 or 310 p*M* PAD4 (Cayman) ± 50 µ*M* GSK484 (Sigma) in citrullination buffer (50 m*M* Tris–HCl pH 7.6, 1 m*M* CaCl_2_, 200 m*M* NaCl and 2 m*M* DTT) in a 384-well Nunc MaxiSorp plate (Thermo Scientific) for 4 h at 37°C and 300 r.p.m. shaking. After overnight storage at 4°C, plates were blocked with 1% BSA in PBS for 2 h at 37°C and 300 r.p.m. shaking, followed by incubation with the primary antibody (anti-citrullinated H3: Cayman, No. 17939) diluted 1:500 in 1% BSA and 0.05% Tween 20 in PBS, and secondary antibody (HRP-conjugated anti-mouse: Sigma, No. A9044) diluted 1:10 000 in 1% BSA and 0.05% Tween 20 in PBS, for 1 h at 37°C and 300 r.p.m. shaking. After every incubation step the plate was washed 3× with 0.05% Tween 20 in PBS. Finally, the secondary antibody was detected through oxidation of 3,3′,5,5′-tetra­methyl­benzidine (Sigma), incubating the reaction for 20 min at 37°C and 300 r.p.m. shaking. The reaction was stopped by adding an equal volume of 0.5 *M* sulfuric acid. The absorbance at 450 nm was measured immediately after (Perkin Elmer Envision 2104).

## 3. Results

### 3.1. Construct design of human PAD6

From the five human PAD homologs, four (PAD1 to PAD4) have been previously purified and structurally characterized by X-ray crystallography (Saijo *et al.*, 2016 ▸; Slade *et al.*, 2015 ▸; Rechiche *et al.*, 2021 ▸; Funabashi *et al.*, 2021 ▸; Arita *et al.*, 2004 ▸). To better understand the relationship between the function and structure of human PAD6, we designed a full-length construct V2-P694 with a TEV-cleavable N-terminal 6×His-GST tag. Two phospho­rylation sites on Ser10 and Ser446 have been identified in human PAD6 (Rose *et al.*, 2012 ▸). In order to understand the impact of these post-translational modifications on its activity, we designed two phospho­mimetic mutations, S10E and S446E, and included them in our final construct. The construct (V2-P694 S10E S446E) was purified to homogeneity, crystallized and characterized biochemically.

### 3.2. PAD6 shows no citrullination activity on proteins *in vitro*

**Figure 1:**
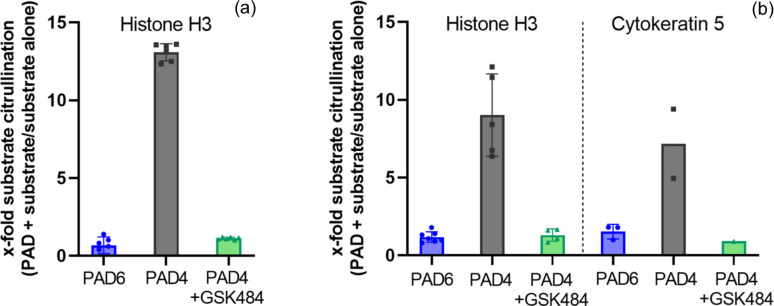
*In vitro* citrullination assays of different substrates. (*a*) ELISA assay for measuring citrullination activity of PAD4 and PAD6 on the histone H3 substrate. PAD6 (blue) and PAD4 (grey) were tested for histone H3 citrullination activity in the presence and absence of 1 µg ml^−1^ histone H3 (two independent experiments with *n* = 3). PAD4 citrullination activity was inhibited by 50 µ*M* GSK484 inhibitor (green). All data were normalized to the citrullination signal of the substrate in the absence of PAD. Error bars indicate the standard deviation. (*b*) On-blot assay for measuring citrullination activity of PAD6 and PAD4 on different substrates. Citrullination of two substrates (histone H3 and cytokeratin 5) was evaluated in presence of PAD6 (blue, *n* = 7 for histone H3, *n* = 3 for cytokeratin 5) or PAD4 (grey, *n* = 5 for histone H3, *n* = 2 for cytokeratin 5) and normalized to the citrullination signal of the substrate in absence of PAD. PAD4 activity was inhibited by 50 µ*M* GSK484 inhibitor (green, *n* = 4 for histone H3, *n* = 1 for cytokeratin 5).

Previous work suggests that PAD6 can citrullinate either histones or cytokeratins in oocytes, as indicated by assays using citrulline-specific antibodies and immunocytochemistry (Esposito *et al.*, 2007 ▸). However, other studies using *in vitro* citrullination assays failed to demonstrate PAD6 activity (Raijmakers *et al.*, 2007 ▸; Taki *et al.*, 2011 ▸). To test the hypothesis that PAD6 is responsible for the citrullination of histone H3 or cytokeratin 5, both potential targets for PAD6 (Schwarz *et al.*, 1995 ▸; Nachat *et al.*, 2005 ▸; Esposito *et al.*, 2007 ▸), we first analyzed the citrullination of histone H3 by an ELISA assay using a specific anti-citrullinated histone H3 antibody. PAD4, which was used as a positive control, clearly increased the citrullination signal for histone H3 [Fig. 1 ▸(*a*)]. The activity of PAD4 was blocked by the addition of the PAD4-specific inhibitor GSK484 (Lewis *et al.*, 2015 ▸). In the presence of PAD6 carrying phospho­mimetic mutations S10E and S446E, the citrullination signal was similar to that of PAD4:GSK484, showing that no detectable citrullination activity could be observed with PAD6 for histone H3. Given the challenge to identify specific antibodies directed against additional citrullinated proteins of interest, we adopted an antibody-independent assay based on the on-blot technology (Hensen *et al.*, 2015 ▸) which allowed us to test additional substrates, such as cytokeratin 5. Data obtained with this orthogonal method clearly confirmed the data obtained with the ELISA method showing the inability of phospho­mimetic PAD6 to citrullinate histone H3. Similar enzymatic citrullination inactivity was detected towards cytokeratin 5 as the substrate for PAD6 [Fig. 1 ▸(*b*) and Fig. S2]. Based on these data, we conclude that, unlike PAD4, PAD6 does not exhibit citrullination activity on the substrates tested *in vitro*.

### 3.3. Tertiary and quaternary structure of PAD6

**Figure 2:**
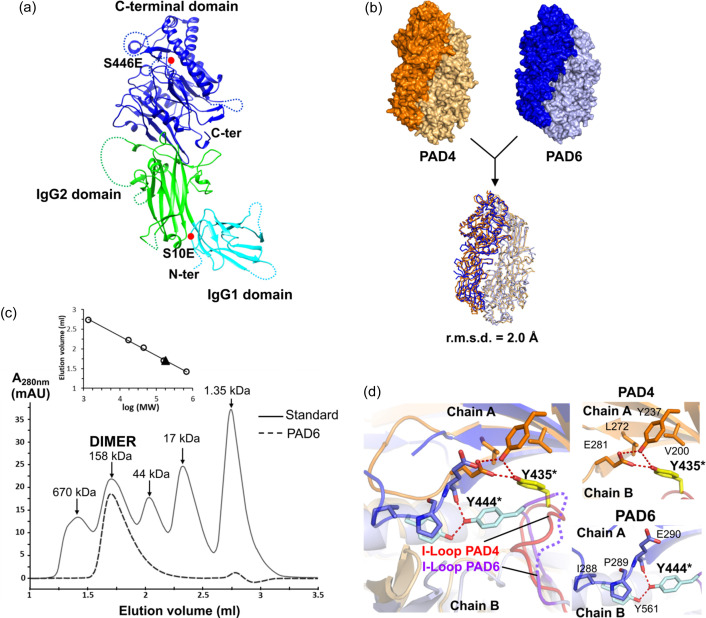
Overall structure of PAD6. (*a*) Domain organization of PAD6. The N-terminal IgG1, N-terminal IgG2 and the C-terminal catalytic domains are coloured in cyan, green and blue, respectively. (*b*) Model of the PAD6 dimer constructed with the symmetry mate of the crystal lattice. Overlay and comparison with the PAD4 dimer and r.m.s.d. calculated from PDB entry 2dew. (*c*) Analytical size-exclusion chromatography of PAD6 using a Superdex 200 5/150GL column equilibrated with 20 m*M* Tris pH 7.5, 200 m*M* NaCl, 1 m*M* TCEP. The elution volumes for PAD6 and bovine gamma globulin standard (158 kDa) were 1.7 ml for both samples. Inlet: standards in white circles and PAD6 in the black triangle. The molecular weight for PAD6 is estimated at ∼182 kDa. (*d*) Comparison between interacting residues involving the I-loops of PAD4 (orange) or that of PAD6 (blue). The I-loop is coloured red in PAD4 and purple in PAD6. In PAD6, the I-loop contains a disordered fragment (Pro445–Gly449) represented as a dashed line. The equivalent residues Tyr435 (PAD4) and Tyr444 (PAD6) are indicated with a star. Left panel: an overlay between PAD4 and PAD6 focused on the I-loop, showing different interface interactions involving Tyr435 (PAD4) and Tyr444 (PAD6). Right panels: detailed descriptions of the interactions involving Tyr435 in PAD4 (top) and those involving Tyr444 in PAD6 (bottom).

PAD6 crystallized in the *C*2 space group, and the structure was determined to a resolution of 1.7 Å (Table 1 ▸). A single chain is present in the asymmetric unit. The tertiary structure of PAD6 is typical of the PAD family and consists of two consecutive immunoglobulin-like domains (IgG1 and IgG2) followed by a α/β propeller C-terminal domain (Saijo *et al.*, 2016 ▸; Slade *et al.*, 2015 ▸; Funabashi *et al.*, 2021 ▸; Arita *et al.*, 2004 ▸). This C-terminal domain contains the catalytic citrullination site in other PADs (Mondal & Thompson, 2019 ▸) [Fig. 2 ▸(*a*)].

PAD4 was found to form a head-to-tail homodimer, with one monomer being related to another PAD molecule by a crystallographic twofold axis (Arita *et al.*, 2004 ▸), and it has been shown that this dimerization is important for optimal activity (Liu *et al.*, 2011 ▸; Lee *et al.*, 2017 ▸). The crystal structures of PAD2 (Slade *et al.*, 2015 ▸) and PAD3 (Funabashi *et al.*, 2021 ▸; Rechiche *et al.*, 2021 ▸) exhibit the same head-to-tail assembly. Examination of the PAD6 crystal packing showed that PAD6 adopts a nearly identical dimeric arrangement across the crystal packing, with a buried surface of 1986.5 Å^2^ at the interface [Fig. S3(*a*)]. This value suggests that the two molecules are involved in a physiological dimerization rather than a crystal lattice (Janin & Chothia, 1990 ▸). Interestingly, the PAD6 assembly closely resembles the PAD4 dimer, since the superposition gives an r.m.s.d. of 2.0 Å with 982 aligned Cα [Fig. 2 ▸(*b*), Fig. S3(*b*)]. This small r.m.s.d. value is in the range of those obtained after pairwise superimpositions between the different dimeric assemblies of PAD isoenzymes (PAD2, PAD3, PAD4 and PAD6), indicating that this quaternary structure organization is well conserved in all these members of the PAD family. An analytical size-exclusion chromatography assay indicates that PAD6 elutes as a dimer in solution, with a molecular weight calculated at ∼182 kDa [Fig. 2 ▸(*c*)]. In addition, a chemical cross-linking experiment showed a unique band observed at ∼150 kDa, consistent with inter-molecular interactions among PAD6 molecules to form dimers [Fig. S3(*c*)]. Altogether, these data suggest that PAD6 most likely dimerizes in the same way as PAD4 and other dimeric PAD isoenzymes.

The dimeric interface is formed by residues from the three domains [Fig. S3(*d*)]. It has been shown in PAD4 that the hydro­phobic nature of several interfacial residues is important for the dimeric stability (Liu *et al.*, 2011 ▸; Lee *et al.*, 2017 ▸). Notably, hydro­phobic residues are also found at equivalent positions in PAD6. Among these, in PAD4, Tyr435 belongs to the ‘interface-loop’ (I-loop) which critically influences both the dimeric stability and the catalytic activity (Lee *et al.*, 2017 ▸). The Tyr435 residue is conserved in PAD6 (Tyr444). The structure reveals a different conformation and interfacial interactions to those in PAD4. This Tyr435 directly interacts with several residues from the facing monomers as it establishes hydrogen bonds with Glu281 and Tyr237 side chains and is involved in a network of hydro­phobic contacts involving Val200 and Leu272. However, in PAD6, the I-loop is partly disordered and Tyr444 interacts with Tyr561 of the same chain, which in turns establishes hydro­phobic contacts with Ile288–Pro289 from the facing monomer [Fig. 2 ▸(*d*)]. This shows that the dimer interface, while being overall well conserved, reveals subtle differences regarding the I-loop.

### 3.4. PAD6 is calcium-free

**Figure 3:**
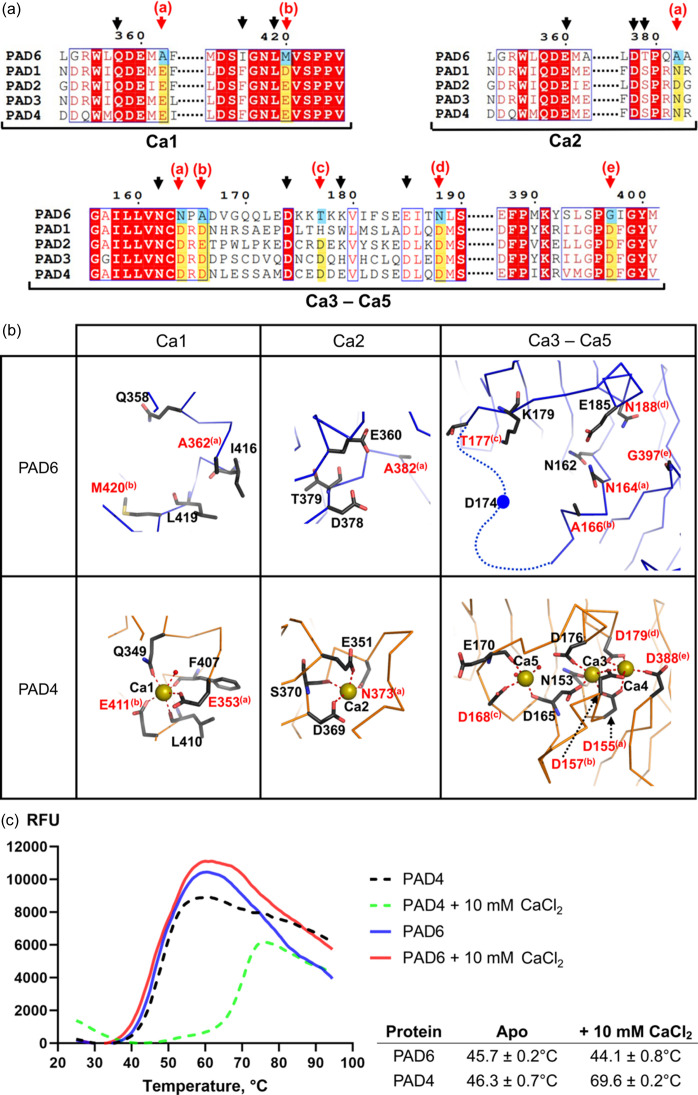
Comparing calcium binding of PAD6 and PAD4. (*a*) Sequence alignment of human PAD6 and other PADs focused on regions containing PAD4 residues involved in Ca1, Ca2, Ca3–5. The arrows point to residues involved in Ca^2+^ coordination in PAD4. The red arrows, with associated letters, highlight residues not conserved and unable to coordinate Ca^2+^ in PAD6. (*b*) Close-up views of PAD6 regions equivalent to Ca^2+^-binding sites in PAD4. Below, PAD4 Ca^2+^-binding sites are shown for reference (PDB entry 2dew). The yellow spheres represent the Ca^2+^ ions in PAD4. Residues that differ between the two proteins for which the side chain cannot coordinate Ca^2+^ in PAD6 are highlighted in red (equivalent positions between PAD6 and PAD4 are indicated with letters in brackets). (*c*) Thermal stability profiles of PAD4 and PAD6 in the presence of 0 or 10 m*M* CaCl_2_. The table provides the average protein melting temperatures (determined as the inflection point of the thermal transition) and the standard deviation from triplicate measurements.

Previous studies on PAD4 revealed the capacity of the enzyme to bind five Ca^2+^ ions cooperatively to transition to the active conformation (Arita *et al.*, 2004 ▸; Liu *et al.*, 2011 ▸). The Ca1 and Ca2 are located in the C-terminal catalytic domain and Ca^2+^ binding at these sites is crucial to shape the substrate-binding site and assist the catalysis (Arita *et al.*, 2004 ▸). Ca3–Ca5 are located further from the active site in the IgG2 domain. Although not essential for activity, Ca^2+^ binding at these sites enhances PAD4 catalytic efficiency (Liu *et al.*, 2013 ▸). PAD1–3 bind calcium at equivalent sites to PAD4 except PAD1, which lacks the binding site for Ca5 (Saijo *et al.*, 2016 ▸; Slade *et al.*, 2015 ▸; Rechiche *et al.*, 2021 ▸). The sequence examination shows that many of the acidic (seven Asp or Glu) and polar (one Asn) residues involved in the Ca^2+^ coordination in PAD4 are not conserved in PAD6 [Fig. 3 ▸(*a*)].

Fig. 3 ▸(*b*) maps the corresponding residues in the PAD6 crystal structure. The regions equivalent to the five Ca^2+^-binding sites in PAD4 are more exposed to solvent than in Ca^2+^-bound PAD4, and they are occupied by water molecules. Within the region equivalent to PAD4 Ca3–Ca5, the dis­ordered segment 170–176 also illustrates its flexibility.

To support the sequence and structural analyses, we performed thermal shift assay to examine the effect of calcium to PAD4 and PAD6. We observed that the addition of 10 m*M* CaCl_2_ significantly increases the melting temperature of PAD4 [Fig. 3 ▸(*c*)], from *T*_m_ = 46.8°C ± 0.7 to *T*_m_ = 69.6°C ± 0.2, indicating that the conformational stability of PAD4 is dependent on Ca^2+^ ions. Conversely, PAD6 was not thermostabilized in the presence of 10 m*M* CaCl_2_. Additionally, isothermal titration calorimetry (ITC) experiments provide further evidence that PAD6 is unlikely to bind Ca^2+^ in contrast to PAD4 (Fig. S4). Altogether, the present analysis suggests that PAD6 is unlikely to coordinate calcium ions.

### 3.5. Inactive form of PAD6

**Figure 4:**
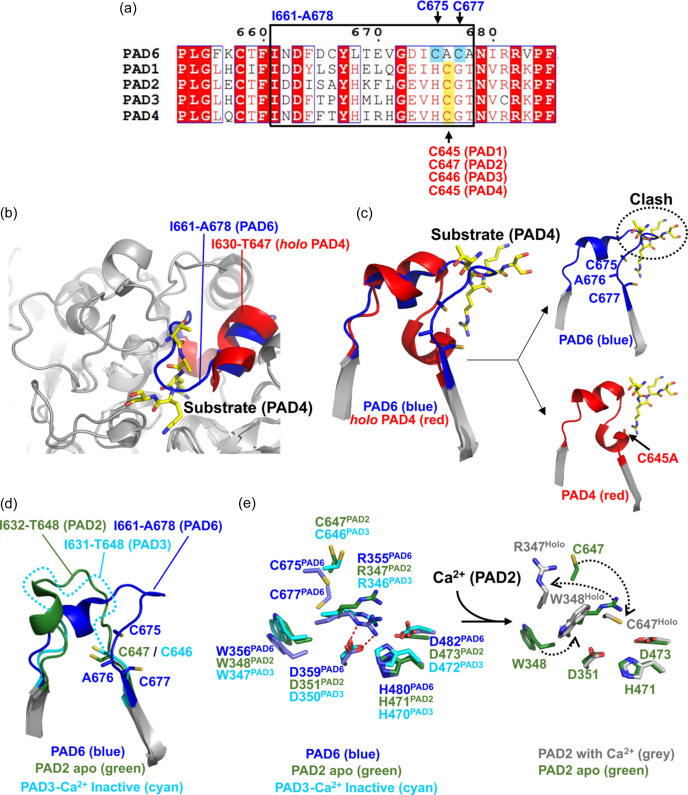
PAD6 structural analysis of the C-terminal domain. (*a*) Sequence alignment between five human PADs, focusing on the PAD6 loop I661–A678. Within this loop, the active or putative active cysteines are underlined and labelled: blue for PAD6, red for the other PADs. (*b*) Superimposition between structures of PAD6 and PAD4 in complex with substrate (PDB entry 2dew). The substrate is in yellow stick representation. (*c*) Same superimposition as in (*b*), showing a close-up view of the segment containing the I661–A678 loop and the β-strands connected by this loop, with the equivalent segment in PAD4 and the substrate. This view shows that the conformation of the PAD6 I661–A678 loop (blue) is different from the equivalent loop in PAD4 (I630–T647, red), and occludes the active site, so that the substrate would clash if positioned as in PAD4 (right panels). (*d*) PAD6 I661–A678 loop in blue superimposed with the equivalent segment of the *apo* PAD2 structure in green (PDB entry 4n20) and the inactive Ca^2+^-PAD3 in cyan (PDB entry 7d8n). Flexible loop I631–T648 (PAD3) represented by a dashed line. (*e*) Similarities in the active sites of the PAD6 structures and the *apo* PAD2 and non-productive form of Ca^2+^-bound PAD3, showing that equivalent Arg residues (Arg355 in PAD6, Arg347 in PAD2 and Arg346 in PAD3) occupy the active site, in lieu of the substrate. Ca^2+^ binding triggers the displacement of Arg347 in *holo* PAD2 (PDB entry 4n2c) together with other residues to shape a functional active site (right panel).

In the current *apo* PAD6 structure, the loops surrounding the putative active site could be entirely traced from the electron density [Fig. S5(*a*)]. In the PAD6 sequence, Ala676 is found at a position equivalent to the active cysteine Cys645 in PAD4, which is conserved in all other PAD isoenzymes [Fig. 4 ▸(*a*)]. Thus, this position cannot serve as a potential catalysis of citrullination in PAD6. However, Ala676 is flanked by two cysteines (Cys675 and Cys677) that could potentially act as active residues. In the structure, neither Cys675 nor Cys677 are favourably positioned and oriented for effective reactivity with the substrate in PAD6, and the I661–A678 loop occludes access to either of these cysteines [Figs. 4 ▸(*b*) and 4 ▸(*c*)]. Fig. 4 ▸(*c*) illustrates the differences between PAD6 and the *holo* PAD4 structure in complex with a substrate, the histone H3 N-terminal tail in which Arg8 is the target for citrullination (PDB entry 2dew; Arita *et al.*, 2006 ▸). In the current PAD6 structure, loop I661–A678 would sterically hinder substrate binding as seen in 2dew [Fig. 4 ▸(*c*), top right]. Additionally, while in the *holo* PAD4 structure the active cysteine (which is intentionally mutated to alanine in 2dew) is part of a small α-helix, Ala676 and Cys677 in PAD6 are part of a β-strand [Fig. 4 ▸(*c*), bottom right]. A *B*-factor analysis indicates that loop I661-A678 has low thermal motion compared with other regions of the structure, suggesting that its conformation is relatively stable [Fig. S5(*b*)]. In the *apo* PAD4 structure, (PDB entry 1wd8; Arita *et al.*, 2004 ▸), the equivalent loop (I630-G646) is dis­ordered, as well as the surrounding loops. In the *apo* PAD2 structure (PDB entry4n20; Slade *et al.*, 2015 ▸), the corresponding loop I635–G648 was modelled and exhibited a conformation characteristic of an inactive state, akin to what is observed in PAD6. Notably, in this loop the PAD2 active cysteine (Cys646) is not in a position suitable for catalytic citrullination [Fig. 4 ▸(*d*)]. Additionally, in this structure, Arg347 shields access to the catalytic centre formed by Asp351, His471 and Asp473 by occupying the substrate-binding cleft (Slade *et al.*, 2015 ▸). In PAD6, the residue corresponding to Arg347 of PAD2, Arg355, adopts a similar position in the vicinity of Asp353, His480 and Asp482 [Fig. 4 ▸(*e*)]. The role of Arg355 as a pseudo-substrate is further supported by a direct interaction with Asp359. Interestingly, a non-productive form of Ca^2+^-bound PAD3 has been crystallized (PDB entry 7d8n; Funabashi *et al.*, 2021 ▸), showing a similar configuration of the active site where the equivalent Arg346 shields access to the catalytic centre [Fig. 4 ▸(*e*)].

Altogether, our structure analysis shows that the PAD6 structure corresponds to an inactive form of PAD that resembles the inactive *apo* PAD2 structure. If PAD6 is to be activated to catalyze citrullination of substrates in the physiological environment, it may be achieved through a specific mechanism, independent to Ca^2+^. The activation of PAD6 would involve the displacement of the loop I661–A678 so that the substrate could access the active centre. In our structure, a PEG molecule from the crystallization solution was found in the vicinity of Cys675 and Cys677 [Fig. S5(*c*)], suggesting that this conformation is not rigid, and the putative active site can be accessed by a potential substrate.

## 4. Discussion

In this study, we determined the first structure of human PAD6. The crystal structure revealed that the protein adopts the typical tertiary structure of the PAD family, with conserved N-terminal immunoglobulin-like domains (IgG1 and IgG2) followed by the C-terminal catalytic domain. The crystal packing analysis suggests that the PAD6 quaternary structure is similar to that of PAD4, a dimer organized in a head-to-tail fashion (Arita *et al.*, 2004 ▸). The dimeric assembly of PAD6 was also observed in solution, suggesting the existence of PAD6 dimers under physiological conditions. Interestingly, all the other structurally known PAD isoenzymes adopt the same dimeric assembly, with the exception of PAD1, which has been shown to be a monomeric PAD (Saijo *et al.*, 2016 ▸). The dimerization state of PAD4 enhances the cooperative binding of Ca^2+^ and enzymatic activity (Liu *et al.*, 2011 ▸), with residues at the dimeric interface exerting a long-distance impact on the active site (Lee *et al.*, 2017 ▸). The binding of five calcium ions to PAD4 induces an active conformation essential for catalytic citrullination (Liu *et al.*, 2011 ▸). In contrast to other PAD isoforms, PAD6 is most likely unable to coordinate calcium ions with most of the residues responsible for the Ca^2+^ binding not conserved. Unsurprisingly, PAD6 was found to be enzymatically inactive for citrullination using histone H3 and cytokeratin 5 *in vitro*, substrates expressed in oocytes (Schwarz *et al.*, 1995 ▸). At the structural level, the PAD6 putative active site is in an un­suitable conformation for substrate binding. Key residues are mis-positioned for an efficient substrate binding and citrullination catalysis, similar to the PAD2 inactive state or to the non-productive Ca^2+^-bound PAD3. These observations raise questions of whether other factors are necessary for PAD6 enzymatic activity and, subsequently, the exact nature of PAD6 substrates. Post-translational modification could be one possibility. Indeed, PAD6 can undergo phospho­rylation during the maturation of mouse oocytes (Snow *et al.*, 2008 ▸) and 2 phospho­rylation sites have been identified in human PAD6 (Rose *et al.*, 2012 ▸). The introduction of the phospho­mimetic mutations S10E and S446E did not result in activity. Although one can consider actual phospho­rylation could have a greater impact than these mutations, the localization of these residues far from the active site rather indicates that PAD6 phospho­rylation at these sites is unlikely to influence its enzymatic activity [Fig. S5(*a*)]. Nonetheless, the accessibility and flexibility of these residues were consistent with previous data showing that both phospho­rylated residues are involved in a protein–protein interaction with 14-3-3 (YWHA) (Snow *et al.*, 2008 ▸; Rose *et al.*, 2012 ▸). 14-3-3, a chaperone-like protein, has also recently been localized in the CPLs (Jentoft *et al.*, 2023 ▸). We cannot rule out that interaction with 14-3-3 or any other PAD6 physiological partners could be required to activate an enzymatic activity via an unknown mechanism. The fact that this mechanism would not depend on Ca^2+^ ions sets PAD6 apart within the PAD family but it remains plausible as illustrated by the bacterial PPAD enzymes which do not utilize Ca^2+^ for citrullination (Goulas *et al.*, 2015 ▸; Bielecka *et al.*, 2014 ▸).

Alternatively, PAD6 might possess a non-enzymatic function in the oocyte. Indeed, several studies have shown PAD6 to be involved in CPL formation (Wright *et al.*, 2003 ▸; Esposito *et al.*, 2007 ▸; Jentoft *et al.*, 2023 ▸). CPLs are highly abundant in oocytes and have long been predicted to function as a storage form for maternal contribution of ribosomes and proteins in oocytes to early embryo (Yurttas *et al.*, 2008 ▸; Capco *et al.*, 1993 ▸; Jentoft *et al.*, 2023 ▸). More recently, PAD6 has also been linked to another cytoplasmic complex, the subcortical maternal complex (SCMC) (Li *et al.*, 2008 ▸; Kim *et al.*, 2010 ▸), a multi-protein complex (∼670–2000 kDa) essential for early embryo­genesis in mouse and human (Li *et al.*, 2008 ▸; Zhu *et al.*, 2015 ▸). Though these observations provide evidence of PAD6 being associated to the cellular and multi-protein complexes (*i.e.* CPL and SCMC), further studies are needed to identify the functional mechanism of PAD6 in early embryo development.

## Supplementary Material

Supporting figures. DOI: 10.1107/S2052252524002549/jt5075sup1.pdf

PDB reference: Human PAD6 phospho­mimic mutant V10E/S446E, *apo*, 8ql0

## Figures and Tables

**Table 1:** Data collection and refinement statistics of PAD6

Data collection			
Space group	*C*2		
Unit-cell parameters			
*a*, *b*, *c* (Å)	127.3, 54.3, 106.8		
α, β, γ (°)	90.0, 108.6, 90.0		
	Overall	Inner shell	Outer shell
Resolution (Å)	101.20–1.68	101.20–5.75	1.85–1.68
Total reflections	281455	13968	13322
Unique reflections	41215	2060	2062
*R*_merge_	0.06	0.04	0.85
Mean *I*/σ(*I*)	13.6	35.6	2.0
Completeness: spherical (%)	51.7	99.2	10.1
Completeness: ellipsoidal (%)	78.7	99.2	57.5
Redundancy	6.8	6.8	6.5
CC_1/2_	1.0	1.0	0.8

Refinement			
Resolution (Å)	101.20–1.68		
Number of reflections (test)	41323 (2087)		
*R*_work_/*R*_free_ (%)	19.7/24.1		
Average protein *B*-factor (Å^2^)	34.9		
Number of atoms			
Protein	5049		
Water	304		
Other	51		
RMS deviation			
Bond lengths (Å)	0.010		
Bond angles (°)	1.01		
Ramachandran plot (%)†			
Favoured regions	97.9		
Allowed regions	1.9		
Disallowed regions	0.2		
PDB entry	8ql0		

† As defined in *MolProbity*.

## References

[bb1] Arita, K., Hashimoto, H., Shimizu, T., Nakashima, K., Yamada, M. & Sato, M. (2004). *Nat. Struct. Mol. Biol.* **11**, 777–783. 10.1038/nsmb799 15247907

[bb2] Arita, K., Shimizu, T., Hashimoto, H., Hidaka, Y., Yamada, M. & Sato, M. (2006). *Proc. Natl Acad. Sci. USA*, **103**, 5291–5296. 10.1073/pnas.0509639103 PMC1459348 16567635

[bb3] Begemann, M., Rezwan, F. I., Beygo, J., Docherty, L. E., Kolarova, J., Schroeder, C., Buiting, K., Chokkalingam, K., Degenhardt, F., Wakeling, E. L., Kleinle, S., González Fassrainer, D., Oehl-Jaschkowitz, B., Turner, C. L. S., Patalan, M., Gizewska, M., Binder, G., Bich Ngoc, C. T., Chi Dung, V., Mehta, S. G., Baynam, G., Hamilton-Shield, J. P., Aljareh, S., Lokulo-Sodipe, O., Horton, R., Siebert, R., Elbracht, M., Temple, I. K., Eggermann, T. & Mackay, D. J. G. (2018). *J. Med. Genet.* **55**, 497–504. 10.1136/jmedgenet-2017-105190 PMC6047157 29574422

[bb4] Bielecka, E., Scavenius, C., Kantyka, T., Jusko, M., Mizgalska, D., Szmigielski, B., Potempa, B., Enghild, J. J., Prossnitz, E. R., Blom, A. M. & Potempa, J. (2014). *J. Biol. Chem.* **289**, 32481–32487. 10.1074/jbc.C114.617142 PMC4239603 25324545

[bb5] Boggs, J. M., Rangaraj, G., Koshy, K. M., Ackerley, C., Wood, D. D. & Moscarello, M. A. (1999). *J. Neurosci. Res.* **57**, 529–535. 10440902

[bb6] Bricogne, G., Blanc, E., Brandl, M., Flensburg, C., Keller, P., Paciorek, W., Roversi, P., Sharff, A., Smart, O. S., Vonrhein, C. & Womack, T. O. (2017). *BUSTER* version 2.11.8. Global Phasing Ltd, Cambridge, United Kingdom.

[bb7] Capco, D. G., Gallicano, G. I., McGaughey, R. W., Downing, K. H. & Larabell, C. A. (1993). *Cell Motil. Cytoskeleton*, **24**, 85–99. 10.1002/cm.970240202 8440027

[bb8] Emsley, P., Lohkamp, B., Scott, W. G. & Cowtan, K. (2010). *Acta Cryst.* D**66**, 486–501. 10.1107/S0907444910007493 PMC2852313 20383002

[bb9] Esposito, G., Vitale, A. M., Leijten, F. P., Strik, A. M., Koonen-Reemst, A. M., Yurttas, P., Robben, T. J., Coonrod, S. & Gossen, J. A. (2007). *Mol. Cell. Endocrinol.* **273**, 25–31. 10.1016/j.mce.2007.05.005 17587491

[bb10] Evans, P. (2006). *Acta Cryst.* D**62**, 72–82. 10.1107/S0907444905036693 16369096

[bb11] Evans, P. R. & Murshudov, G. N. (2013). *Acta Cryst.* D**69**, 1204–1214. 10.1107/S0907444913000061 PMC3689523 23793146

[bb12] Funabashi, K., Sawata, M., Nagai, A., Akimoto, M., Mashimo, R., Takahara, H., Kizawa, K., Thompson, P. R., Ite, K., Kitanishi, K. & Unno, M. (2021). *Arch. Biochem. Biophys.* **708**, 108911. 10.1016/j.abb.2021.108911 33971157

[bb13] Goulas, T., Mizgalska, D., Garcia-Ferrer, I., Kantyka, T., Guevara, T., Szmigielski, B., Sroka, A., Millán, C., Usón, I., Veillard, F., Potempa, B., Mydel, P., Solà, M., Potempa, J. & Gomis-Rüth, F. X. (2015). *Sci. Rep.* **5**, 11969. 10.1038/srep11969 PMC4487231 26132828

[bb14] Hensen, S. M. M., Boelens, W. C., Bonger, K. M., van Cruchten, R. T. P., van Delft, F. L. & Pruijn, G. J. M. (2015). *Molecules*, **20**, 6592–6600. 10.3390/molecules20046592 PMC6272700 25875038

[bb15] Janin, J. & Chothia, C. (1990). *J. Biol. Chem.* **265**, 16027–16030. 2204619

[bb16] Jentoft, I. M. A., Bäuerlein, F. J. B., Welp, L. M., Cooper, B. H., Petrovic, A., So, C., Penir, S. M., Politi, A. Z., Horokhovskyi, Y., Takala, I., Eckel, H., Moltrecht, R., Lénárt, P., Cavazza, T., Liepe, J., Brose, N., Urlaub, H., Fernández-Busnadiego, R. & Schuh, M. (2023). *Cell*, **186**, 5308–5327.e25. 10.1016/j.cell.2023.10.003 37922900

[bb17] Jumper, J., Evans, R., Pritzel, A., Green, T., Figurnov, M., Ronneberger, O., Tunyasuvunakool, K., Bates, R., Žídek, A., Potapenko, A., Bridgland, A., Meyer, C., Kohl, S. A. A., Ballard, A. J., Cowie, A., Romera-Paredes, B., Nikolov, S., Jain, R., Adler, J., Back, T., Petersen, S., Reiman, D., Clancy, E., Zielinski, M., Steinegger, M., Pacholska, M., Berghammer, T., Bodenstein, S., Silver, D., Vinyals, O., Senior, A. W., Kavukcuoglu, K., Kohli, P. & Hassabis, D. (2021). *Nature*, **596**, 583–589. 10.1038/s41586-021-03819-2 PMC8371605 34265844

[bb18] Kabsch, W. (2010). *Acta Cryst.* D**66**, 125–132. 10.1107/S0907444909047337 PMC2815665 20124692

[bb19] Kim, B., Kan, R., Anguish, L., Nelson, L. M. & Coonrod, S. A. (2010). *PLoS One*, **5**, e12587. 10.1371/journal.pone.0012587 PMC2935378 20830304

[bb21] Lee, C. Y., Lin, C. C., Liu, Y. L., Liu, G. Y., Liu, J. H. & Hung, H. C. (2017). *Sci. Rep.* **7**, 42662. 10.1038/srep42662 PMC5314407 28209966

[bb22] Lewis, H. D., Liddle, J., Coote, J. E., Atkinson, S. J., Barker, M. D., Bax, B. D., Bicker, K. L., Bingham, R. P., Campbell, M., Chen, Y. H., Chung, C. W., Craggs, P. D., Davis, R. P., Eberhard, D., Joberty, G., Lind, K. E., Locke, K., Maller, C., Martinod, K., Patten, C., Polyakova, O., Rise, C. E., Rüdiger, M., Sheppard, R. J., Slade, D. J., Thomas, P., Thorpe, J., Yao, G., Drewes, G., Wagner, D. D., Thompson, P. R., Prinjha, R. K. & Wilson, D. M. (2015). *Nat. Chem. Biol.* **11**, 189–191. 10.1038/nchembio.1735 PMC4397581 25622091

[bb23] Li, L., Baibakov, B. & Dean, J. (2008). *Dev. Cell*, **15**, 416–425. 10.1016/j.devcel.2008.07.010 PMC2597058 18804437

[bb24] Liu, J., Tan, Z., He, J., Jin, T., Han, Y., Hu, L. & Huang, S. (2021). *J. Assist. Reprod. Genet.* **38**, 1551–1559. 10.1007/s10815-021-02194-1 PMC8266952 34036456

[bb25] Liu, Y. L., Chiang, Y. H., Liu, G. Y. & Hung, H. C. (2011). *PLoS One*, **6**, e21314. 10.1371/journal.pone.0021314 PMC3120853 21731701

[bb26] Liu, Y. L., Tsai, I. C., Chang, C. W., Liao, Y. F., Liu, G. Y. & Hung, H. C. (2013). *PLoS One*, **8**, e51660. 10.1371/journal.pone.0051660 PMC3559651 23382808

[bb27] Mondal, S. & Thompson, P. R. (2019). *Acc. Chem. Res.* **52**, 818–832. 10.1021/acs.accounts.9b00024 PMC6443095 30844238

[bb28] Muth, A., Subramanian, V., Beaumont, E., Nagar, M., Kerry, P., McEwan, P., Srinath, H., Clancy, K., Parelkar, S. & Thompson, P. R. (2017). *J. Med. Chem.* **60**, 3198–3211. 10.1021/acs.jmedchem.7b00274 PMC5477668 28328217

[bb29] Nachat, R., Méchin, M. C., Takahara, H., Chavanas, S., Charveron, M., Serre, G. & Simon, M. (2005). *J. Invest. Dermatol.* **124**, 384–393. 10.1111/j.0022-202X.2004.23568.x 15675958

[bb30] Raijmakers, R., Zendman, A. J., Egberts, W. V., Vossenaar, E. R., Raats, J., Soede-Huijbregts, C., Rutjes, F. P., van Veelen, P. A., Drijfhout, J. W. & Pruijn, G. J. (2007). *J. Mol. Biol.* **367**, 1118–1129. 10.1016/j.jmb.2007.01.054 17303166

[bb31] Rechiche, O., Lee, T. V. & Lott, J. S. (2021). *Acta Cryst.* F**77**, 334–340. 10.1107/S2053230X21009195 PMC8488854 34605437

[bb32] Rose, R., Rose, M. & Ottmann, C. (2012). *J. Struct. Biol.* **180**, 65–72. 10.1016/j.jsb.2012.05.010 22634725

[bb33] Saijo, S., Nagai, A., Kinjo, S., Mashimo, R., Akimoto, M., Kizawa, K., Yabe-Wada, T., Shimizu, N., Takahara, H. & Unno, M. (2016). *J. Mol. Biol.* **428**, 3058–3073. 10.1016/j.jmb.2016.06.018 27393304

[bb34] Schwarz, S. M., Gallicano, G. I., McGaughey, R. W. & Capco, D. G. (1995). *Mech. Dev.* **53**, 305–321. 10.1016/0925-4773(95)00440-8 8645598

[bb35] Senshu, T., Akiyama, K., Ishigami, A. & Nomura, K. (1999). *J. Dermatol. Sci.* **21**, 113–126. 10.1016/s0923-1811(99)00026-2 10511480

[bb36] Slade, D. J., Fang, P., Dreyton, C. J., Zhang, Y., Fuhrmann, J., Rempel, D., Bax, B. D., Coonrod, S. A., Lewis, H. D., Guo, M., Gross, M. L. & Thompson, P. R. (2015). *Am. Chem. Soc. Chem. Biol.* **10**, 1043–1053. 10.1021/cb500933j PMC4569063 25621824

[bb37] Snow, A. J., Puri, P., Acker-Palmer, A., Bouwmeester, T., Vijayaraghavan, S. & Kline, D. (2008). *Biol. Reprod.* **79**, 337–347. 10.1095/biolreprod.108.069328 PMC2575841 18463355

[bb38] Suzuki, A., Yamada, R., Chang, X., Tokuhiro, S., Sawada, T., Suzuki, M., Nagasaki, M., Nakayama-Hamada, M., Kawaida, R., Ono, M., Ohtsuki, M., Furukawa, H., Yoshino, S., Yukioka, M., Tohma, S., Matsubara, T., Wakitani, S., Teshima, R., Nishioka, Y., Sekine, A., Iida, A., Takahashi, A., Tsunoda, T., Nakamura, Y. & Yamamoto, K. (2003). *Nat. Genet.* **34**, 395–402. 10.1038/ng1206 12833157

[bb39] Taki, H., Gomi, T., Knuckley, B., Thompson, P. R., Vugrek, O., Hirata, K., Miyahara, T., Shinoda, K., Hounoki, H., Sugiyama, E., Usui, I., Urakaze, M., Tobe, K., Ishimoto, T., Inoue, R., Tanaka, A., Mano, H., Ogawa, H. & Mori, H. (2011). *Adv. Biosci. Biotechnol.* **02**, 304–310.

[bb40] Tickle, I. J., Flensburg, C., Keller, P., Paciorek, W., Sharff, A., Vonrhein, C. & Bricogne, G. (2018). *STARANISO*. Global Phasing Ltd, Cambridge, United Kingdom.

[bb41] Verheul, M. K., van Veelen, P. A., van Delft, M. A. M., de Ru, A., Janssen, G. M. C., Rispens, T., Toes, R. E. M. & Trouw, L. A. (2018). *Autoimmun. Rev.* **17**, 136–141. 10.1016/j.autrev.2017.11.017 29203292

[bb42] Vonrhein, C., Flensburg, C., Keller, P., Sharff, A., Smart, O., Paciorek, W., Womack, T. & Bricogne, G. (2011). *Acta Cryst.* D**67**, 293–302. 10.1107/S0907444911007773 PMC3069744 21460447

[bb43] Vossenaar, E. R., Zendman, A. J., van Venrooij, W. J. & Pruijn, G. J. (2003). *BioEssays*, **25**, 1106–1118. 10.1002/bies.10357 14579251

[bb44] Winn, M. D., Ballard, C. C., Cowtan, K. D., Dodson, E. J., Emsley, P., Evans, P. R., Keegan, R. M., Krissinel, E. B., Leslie, A. G. W., McCoy, A., McNicholas, S. J., Murshudov, G. N., Pannu, N. S., Potterton, E. A., Powell, H. R., Read, R. J., Vagin, A. & Wilson, K. S. (2011). *Acta Cryst.* D**67**, 235–242. 10.1107/S0907444910045749 PMC3069738 21460441

[bb45] Wright, P. W., Bolling, L. C., Calvert, M. E., Sarmento, O. F., Berkeley, E. V., Shea, M. C., Hao, Z., Jayes, F. C., Bush, L. A., Shetty, J., Shore, A. N., Reddi, P. P., Tung, K. S., Samy, E., Allietta, M. M., Sherman, N. E., Herr, J. C. & Coonrod, S. A. (2003). *Dev. Biol.* **256**, 73–88. 10.1016/s0012-1606(02)00126-4 12654293

[bb46] Xu, Y., Shi, Y., Fu, J., Yu, M., Feng, R., Sang, Q., Liang, B., Chen, B., Qu, R., Li, B., Yan, Z., Mao, X., Kuang, Y., Jin, L., He, L., Sun, X. & Wang, L. (2016). *Am. J. Hum. Genet.* **99**, 744–752. 10.1016/j.ajhg.2016.06.024 PMC5010645 27545678

[bb47] Yurttas, P., Vitale, A. M., Fitzhenry, R. J., Cohen-Gould, L., Wu, W., Gossen, J. A. & Coonrod, S. A. (2008). *Development*, **135**, 2627–2636. 10.1242/dev.016329 PMC2708103 18599511

[bb48] Zhu, K., Yan, L., Zhang, X., Lu, X., Wang, T., Yan, J., Liu, X., Qiao, J. & Li, L. (2015). *Mol. Hum. Reprod.* **21**, 320–329. 10.1093/molehr/gau116 25542835

