# Supplementary material for: Crystal structure of human peptidylarginine deiminase type VI (PAD6) provides insights into its inactivity
Source: IUCrJ. 2024 Apr 24;11(Pt 3):395–404. doi: 10.1107/S2052252524002549 (PMC11067741; doi:10.1107/S2052252524002549)
Supplement: Supplementary file 1 [file m-11-00395-sup1.pdf]

IUCrJ

Volume 11 (2024)

Supporting information for article:

Crystal structure of human peptidylarginine deiminase type VI (PAD6) provides insights into its inactivity

Fanomezana Ranaivoson, Rieke Bande, Isabell Cardaun, Antonio De Riso, Annette Gaertner, Pui Loke, Christina Reinisch, Prasuna Vogirala and Edward Beaumont

PAD6:

MGSHHHHHHGSSPILGYWKIKGLVQPTRLLLEYLEEKYEEHLYERDEGDKWRNKKFELGLEFPNLPYYIDGDVKL
TQSMAIIRYIADKHNMLGGCPKERAEISMLEGAVLDIRYGVSRIAYSKDFETLKVDFLSKLPEMLKMFEDRLCHKT
YLNGDHVTHPDFMLYDALDVVLYMDPMCLDAFPKLVCFKKRIEAIPQIDKYLKSSKYIAWPLQGWQATFGGGDH
PPKSDENLYFQSVSVEGRAMEFQSIIHLSLDSPVHAVCVLGTEICLDLSGCAPQKCQCFTIHGSGRVLIDVANTVISE
KEDATIWWPLSDPTYATVKMTSPSPSVDADKVSVTYYGPNEDAPVGTAVLYLTGIEVSLEVDIYRNGQVEMSSDK
QAKKKWIWGPSGWGAILLVNCNPADVGQQLEDKKTKKVIFSEEITNLSQMTLNVQGPSCILKKYRLVLHTSKEESK
KARVYWPQKDNSSTFELVLGPDQHAYTLALLGNHLKETFYVEAIAFPSAEFSGLISYSVSLVEESQDPSIPETVLYKD
TVVFRVAPCVFIPCTQVPLEVYLCRELQLQGFVDTVTKLSEKSNSQVASVYEDPNRLGRWLQDEMAFCYTQAPHK
TTSLILDTPQAADLDEFPMKYSLSPGIGYMIQDTEDHKVASMDSIGNLMVSPPVKVQGKEYPLGRVLIGSSFYPEAE
GRAMSKTLRDFLYAQQVQAPVELYSDWLMTGHVDEFMCFIPTDDKNEGKKGFLLLLASPSACYKLFREKQKEGY
GDALLFDELRADQLLSNGREAKTIDQLLADESLKKQNEYVEKCIHLNRDILKTELGLVEQDIIEIPQLFCLEKLTNIPS
DQQPKRSFARPYFPDLLRMIVMGKNLGIPKPFGPQIKGTCCLEEKICCLLEPLGFKCTFINDFDCYLTEVGDICACANI
RRVPFAFKWWKMVP

PAD4:

MSPILGYWKIKGLVQPTRLLLEYLEEKYEEHLYERDEGDKWRNKKFELGLEFPNLPYYIDGDVKLTQSMAIIRYIAD
KHNMLGGCPKERAEISMLEGAVLDIRYGVSRIAYSKDFETLKVDFLSKLPEMLKMFEDRLCHKTYLNGDHVTHPD
FMLYDALDVVLYMDPMCLDAFPKLVCFKKRIEAIPQIDKYLKSSKYIAWPLQGWQATFGGGDHPPKSDLEVLFQG
PLGSPEFMAQGTLIRVTPEQPTHAVCVLGTLTQLDICSSAPEDCTSFSINASPGVVVDIAHSPPAKKKSTGSSTWPLDP
GVEVTLTMKAASGSTGDQKVQISYYGPKTPPVKALLYLTAVEISLCADITRTGKVKPTRAVKDQRTWTWGPCGQG
AILLVNCDRDNLESSAMDCEDDEVLDSEDLQDMSLMTLSTKTPKDFFTNHTLVLHVARSEMDKVRVFQATRGKLS
SKCSVVLGPKWPSHYLMVPGGKHNMDFYVEALAFPDTDFPGLITLTISLLDTSNLELPEAVVFQDSVVFRVAPWIM
TPNTQPPQEVYACSIFENEDFLKSVTTLAMKAKCKLTICPEEENMDDQWMQDEMEIGYIQAPHKTLPVVFDSPRNR
GLKEFPIKRVMGPDFGYVTRGPQTGGISGLDSFGNLEVSPPVTVRGKEYPLGRILFGDSCYPSNDSRQMHQALQDFL
SAQQVQAPVKLYSDWLSVGHVDEFLSFVPAPDRKGFRLLLASPRSCYKLFQEQQNEGHGEALLFEGIKKKKQQKIK
NILSNKTLREHNSFVERCIDWNRELLKRELGLAESDIIDIPQLFKLKEFSKAEAFFPNMVNMLVLGKHLGIPKPFGPVI
NGRCCLEEKVCSLLEPLGLQCTFINDFFTYHIRHGEVHCGTNVRRKPFSFKWWNMVP

Figure S1 Sequences of the protein constructs expressed for this study.

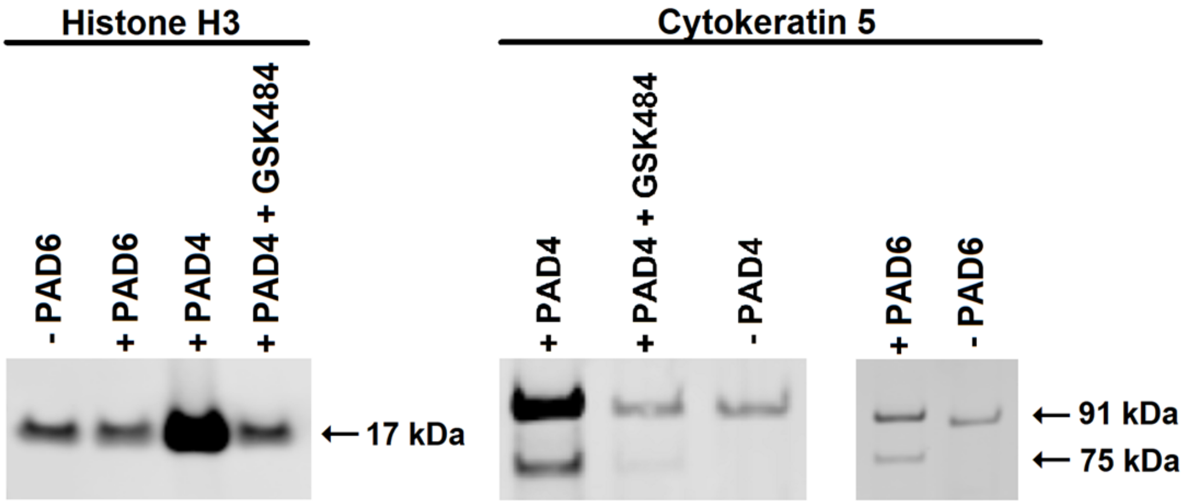

Figure S2 PAD4 but not PAD6 catalyzed the citrullination of different substrates. On-blot assay for measuring citrullination activity of PAD4 and PAD6 on different substrates. Citrullination of two substrates (histone H3 and cytokeratin 5) was evaluated in presence of PAD4 or PAD6. PAD4 activity was inhibited by 50 μM GSK484 inhibitor. A clear citrullination of histone H3 and cytokeratin 5 by Pad4 was seen. Pad6 did not increase citrullination of histone H3 and cytokeratin 5 over the background. The bands at 75 kDa represent PAD4 or PAD6.

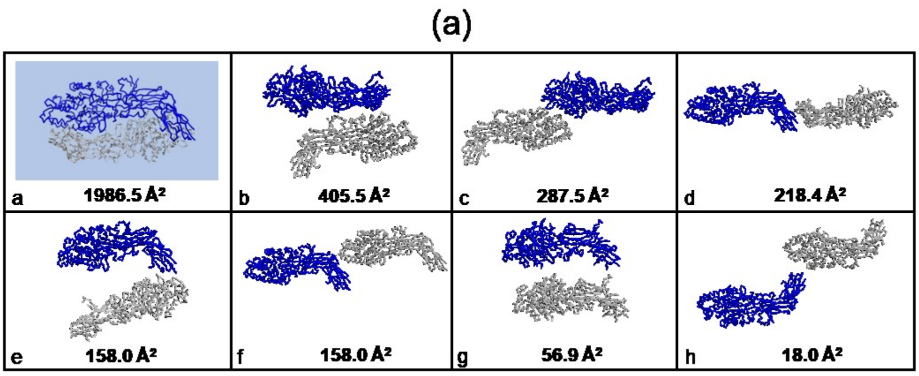

(b)

	PAD2	PAD3	PAD4	PAD6
PAD2	-	3.44 (1083)	2.78 (986)	2.19 (948)
PAD3		-	2.48 (1004)	3.08 (971)
PAD4			-	1.96 (982)
PAD6				-

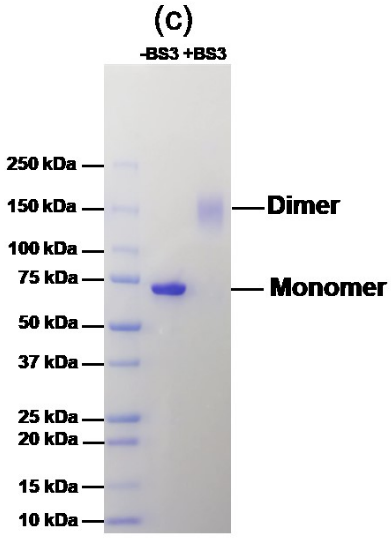

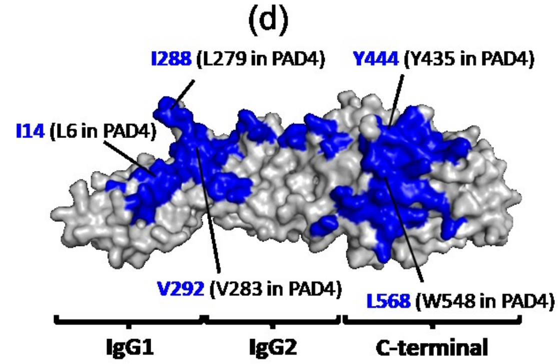

Figure S3 Dimerization of PAD6. (a) Different pairwise combinations of the PAD6 symmetry mates in the crystal lattice and their interface area analyzed with PISA (Krissinel & Henrick, 2007). a, in blue, represents the head-to-tail PAD6 homodimer with 1986.5 Å2 interface area. (b) r.m.s.d. values from pairwise superpositions of the structurally known human PAD dimeric assemblies. In each case, the number of aligned Cα is indicated in brackets. In addition to PAD6, the compared structures were with the following PDB IDs: 4N20 (PAD2), 7D4Y (PAD3) and 1WD8 (PAD4). (c) SDS-PAGE after chemical crosslinking assay on PAD6 using bis(sulfosuccinimidyl)suberate (BS3). After 4hrs reaction, no band corresponding to a monomer could be detected, but a band with a molecular weight consistent with a dimer (~150 kDa) is visible. Prior to chemical cross-linking, PAD6 was buffer-exchanged with 20 mM HEPES pH 7.8; 300 mM NaCl; 0.5 mM TCEP. 50-times molar excess of BS3 (Thermo Scientific) was added to 40 µM PAD6. After 4 hours incubation at room temperature (~22 ˚C), 2.5 ug protein was taken from this solution or a control solution (PAD6 without cross-linker) and added to 2X reducing SDS-PAGE loading buffer (62.5 mM Tris-HCl, pH 6.8, 25% glycerol, 2% SDS 0.01% Bromophenol Blue, 1mM TCEP) (Bio-Rad) to quench the cross-linking reaction. (d) Surface representation of PAD6 structure highlighting residues buried in the dimeric interface (blue). Six interfacial hydrophobic residues are indicated with their equivalent in PAD4 (in brackets). Hydrophobicity of these residues have been shown to be important for PAD4 dimerization (Liu *et al.*, 2011, Lee *et al.*, 2017).

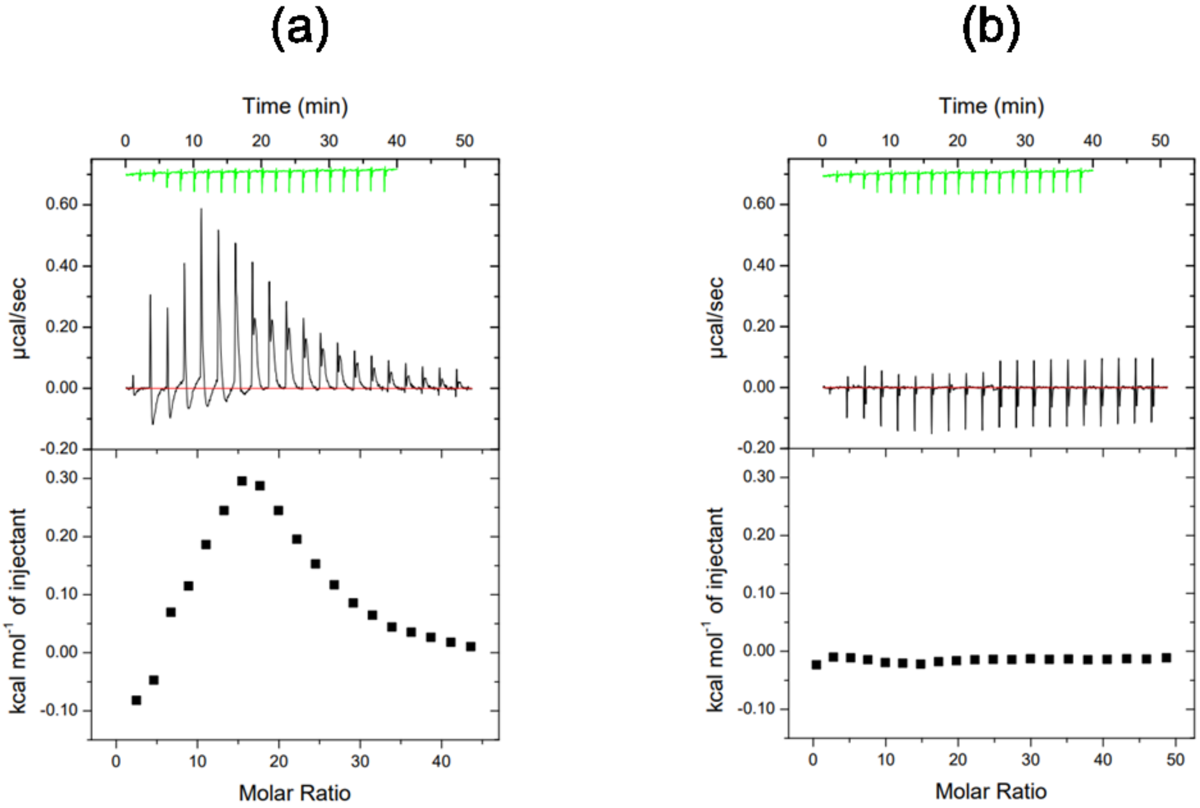

Figure S4 Isothermal titration calorimetry profiles for Ca^{2+} to PAD4 and PAD6 at 25°C in 20 mM Tris-HCl pH 7.5, 300 mM NaCl, 0.5 mM TCEP and 5 % v/v glycerol. The upper panels show the calorimetric titrations with 2 µl injections of 20 mM $CaCl_2$ into 100 µM of protein (a) PAD4. (b) PAD6. The control $CaCl_2$ into buffer is represented in green. The lower panels display the integrated heat values from the upper panels as a function of the molar ratio of Ca^{2+} to PAD in the reaction cell. Ca^{2+} titration to PAD4 exhibits a complex behaviour involving both exothermic and endothermic processes. The data were of insufficient quality to determine the Ca^{2+} binding parameters with certainty.

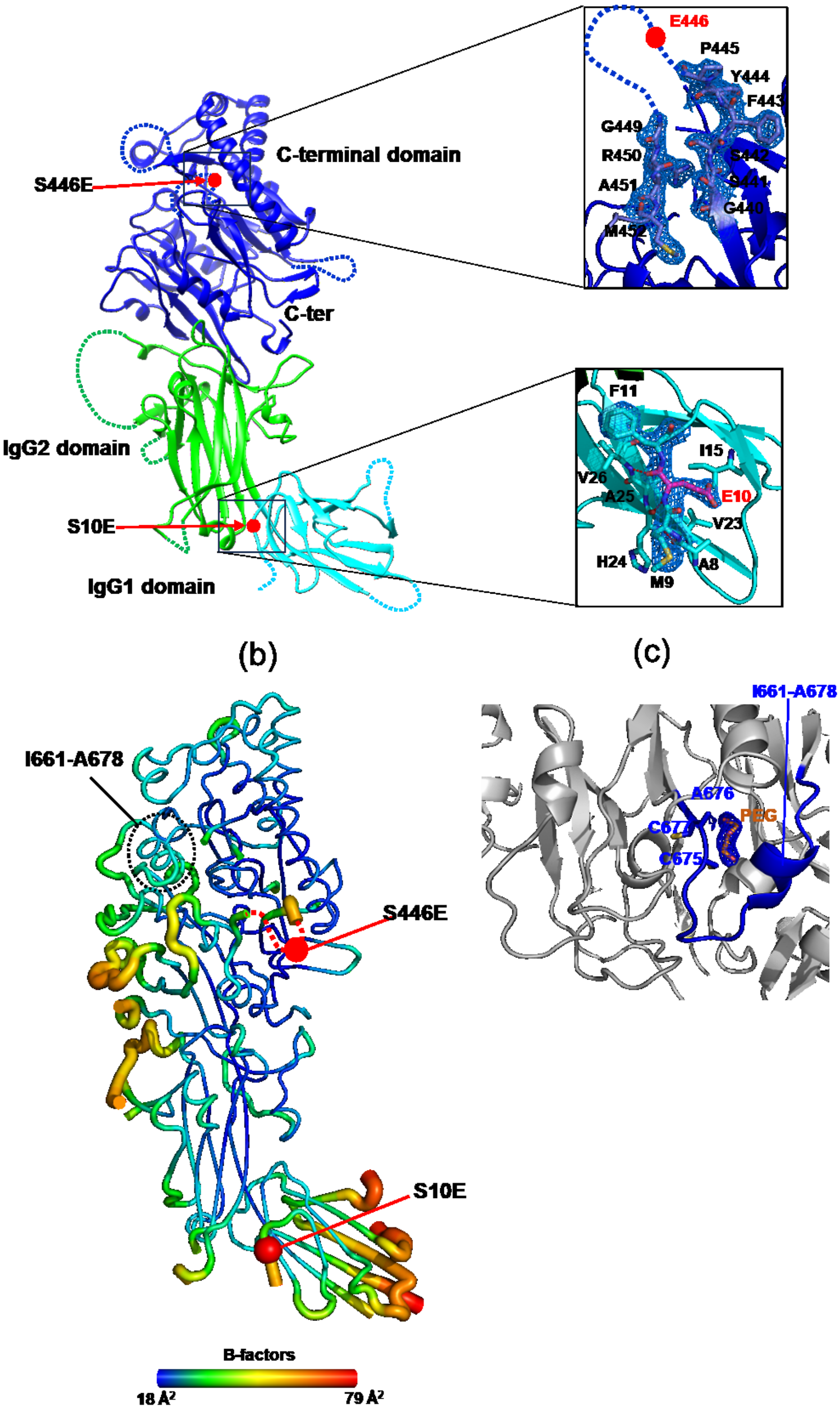

Figure S5 Different representations and views of PAD6 structure. (a) Cartoon representation as in Figure 2a, with a zoom in the region of the phosphomimetic Glu mutations (S10E) and (S446E). Electron density maps of residues in these regions are shown (2mFo-DFc, contour level 1.0 σ). Phosphomimetic S10E is part of the β-sheet in the IgG1 domain and faces 2 hydrophobic residues, I15 and V23. S446E is located on a disordered loop. (b) Representation according to B-factors of α-carbons**.** The two phosphomimetic are represented as red sphere; S446E being in a disordered loop, and not included in the final model. The putative active site loop I661-A678 is also indicated with a dashed circle. (c) An overall view of the putative active site region in PAD6. Highlighted in blue the I661-A678 loop containing the Cys675 and Cys677, that could act as catalytic residues in PAD6.
